# Pharmacological targeting of c-FLIP_L_ and Bcl-2 family members promotes apoptosis in CD95L-resistant cells

**DOI:** 10.1038/s41598-020-76079-1

**Published:** 2020-11-30

**Authors:** Corinna König, Laura K. Hillert-Richter, Nikita V. Ivanisenko, Vladimir A. Ivanisenko, Inna N. Lavrik

**Affiliations:** 1 Translational Inflammation Research, Medical Faculty, CDS, Otto von Guericke University Magdeburg, 39106 Magdeburg, Germany; 2 Federal Research Center Institute of Cytology and Genetics, Siberian Branch of Russian Academy of Sciences, Prospekt Lavrentyeva 10, Novosibirsk, 630090 Russia; 3 Kurchatov Genomics Center, Institute of Cytology and Genetics, Siberian Branch of Russian Academy of Sciences, Prospekt Lavrentyeva 10, Novosibirsk, 630090 Russia

**Keywords:** Cell death, Computational chemistry

## Abstract

The development of efficient combinatorial treatments is one of the key tasks in modern anti-cancer therapies. An apoptotic signal can either be induced by activation of death receptors (DR) (extrinsic pathway) or via the mitochondria (intrinsic pathway). Cancer cells are characterized by deregulation of both pathways. Procaspase-8 activation in extrinsic apoptosis is controlled by c-FLIP proteins. We have recently reported the small molecules FLIPinB/FLIPinBγ targeting c-FLIP_L_ in the caspase-8/c-FLIP_L_ heterodimer. These small molecules enhanced caspase-8 activity in the death-inducing signaling complex (DISC), CD95L/TRAIL-induced caspase-3/7 activation and subsequent apoptosis. In this study to increase the pro-apoptotic effects of FLIPinB/FLIPinBγ and enhance its therapeutic potential we investigated costimulatory effects of FLIPinB/FLIPinBγ in combination with the pharmacological inhibitors of the anti-apoptotic Bcl-2 family members such as ABT-263 and S63845. The combination of these inhibitors together with FLIPinB/FLIPinBγ increased CD95L-induced cell viability loss, caspase activation and apoptosis. Taken together, our study suggests new approaches for the development of combinatorial anti-cancer therapies specifically targeting both intrinsic and extrinsic apoptosis pathways.

## Introduction

Apoptosis is a form of programmed cell death that is essential for all multicellular organisms. An apoptotic signal can be induced by a variety of factors, including death receptor (DR) activation^1^. The apoptotic DR signaling cascade is triggered by the activation of a corresponding DR, CD95/Fas or TRAIL-R1/2, which results in the formation of a death-inducing signaling complex (DISC). DISC comprises DR, FADD, procaspases-8, -10 and c-FLIP, serving as a central platform for procaspase-8 activation, which subsequently initiates an apoptotic response^2^. The molecular architecture of the DISC can be characterized as a framework of strictly-defined interactions between death domains (DDs) and death effector domains (DEDs). Procaspase-8 at the DISC forms DED filaments via DED interactions which serve as a platform for dimerization and its subsequent activation^3–5^.

**Figure 1:**
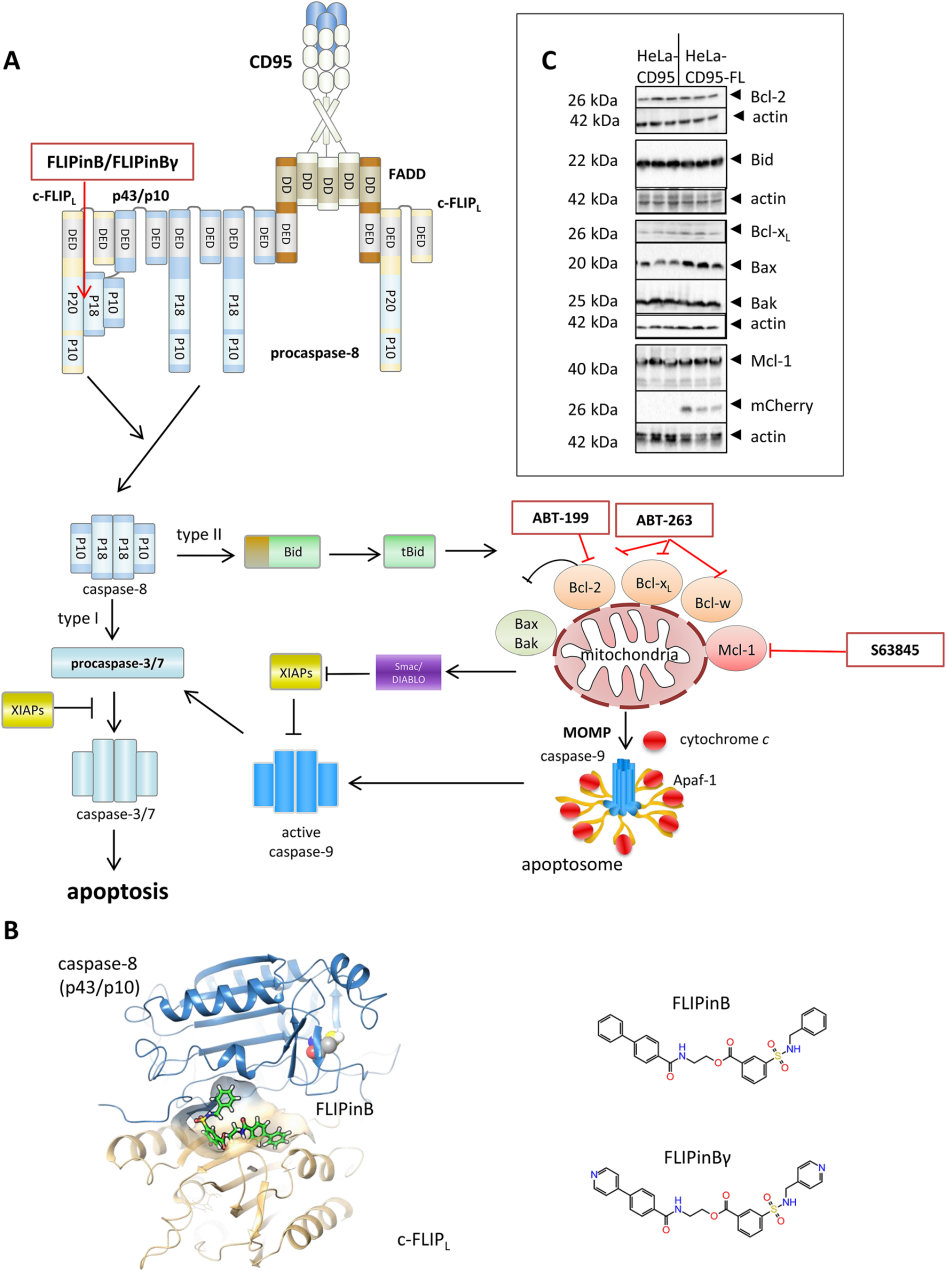
Schematic representation of the inhibitors influencing the CD95 pathway. (**A**) CD95L triggers the DISC assembly comprising FADD, procaspase-8 and c-FLIP. FLIPinB and FLIPinBγ promote caspase-8 activity at the DISC by binding to caspase-8(p43/p10)/c-FLIP_L_ heterodimer at the DED filaments. This leads to an increased caspase-8 activation. Caspase-8 activates caspase-3 which leads to apoptosis. In type II cells caspase-8 cleaves Bid to tBid which leads to activation of pro-apoptotic members of the Bcl-2 family and inhibition of anti-apoptotic members of the Bcl-2 family. This is followed by MOMP and release of cytochrome *c* and the formation of the apoptosome which in turn leads also to caspase-3 activation and apoptosis. ABT-199 blocks Bcl-2 by binding to it while ABT-263 can block Bcl-2, Bcl-x_L_ and Bcl-w. S63845 is an inhibitor of Mcl-1. (**B**) On the left side the caspase-8(p43/p10)/c-FLIP_L_ heterodimer is shown with caspase-8(p43/p10) in blue and c-FLIP_L_ in brown. FLIPinB is shown in green. The active site cysteine of caspase-8 is shown as spheres. On the right side the structures of FLIPinB and FLIPinBγ are shown. (**C**) Total cell lysates of HeLa-CD95 cells (CD95 overexpressing cells) and HeLa-CD95-FL cells (c-FLIP_L_ overexpressing HeLa-CD95 cells) were analyzed by Western Blot using the indicated antibodies. The samples were loaded on four different gels marked by the white space between the corresponding Western Blots. Actin served as a loading control for each gel.

Downstream of the DISC formation, the apoptotic signal transduction is controlled at several levels. Two types of cells are described^6^. Type I cells are characterized by high numbers of the CD95 DISC, and, correspondingly, high amounts of active caspase-8, that is leading to the activation of caspase-3 and apoptosis induction (Fig. 1A). In type II cells a lower number of the CD95 DISC is formed and the apoptotic signal requires an additional amplification loop that involves cleavage of Bid by procaspase-8 and translocation of truncated Bid (tBid) to mitochondria (Fig. 1A). This is followed by mitochondrial outer membrane permeabilization (MOMP), cytochrome *c* release from mitochondria, subsequent apoptosome formation, resulting in procaspase-9 and then caspase-3 activation.

The apoptosis induction in type II cells could be blocked by anti-apoptotic Bcl-2 family members. In the recent years, an enourmous progress has been achieved in targeting these proteins and thereby promoting apoptosis. In particular, the specific inhibitors of the anti-apoptotic Bcl-2 family members have been developed including small molecules such as ABT-263/navitoclax, ABT-199/venetoclax and S63845 (Fig. 1A). ABT-263 blocks Bcl-2, Bcl-x_L_ and Bcl-w, ABT-199 targets only Bcl-2, while S63845 is an inhibitor of Mcl-1^7–10^. These inhibitors are already in clinical trials and made a significant contribution to the development of novel anti-cancer therapies^11,12^. The Bcl-2 inhibitor ABT-199 has been approved for treatment of refractory chronic lymphocytic leukemia and together with inhibitors of Bcl-x_L_ and Mcl-1 is being tested in diverse malignancies^13^.

c-FLIP proteins are main inhibitors of procaspase-8 activation at the DISC and DED filaments^14^. Three c-FLIP isoforms, including Long (L), Short (S), and Raji (R), i.e., c-FLIP_L_, c-FLIP_S_, and c-FLIP_R_ have been characterized so far^15–18^. All three isoforms possess two DED domains at their N-terminus. c-FLIP_L_ also contains catalytically-inactive caspase-like domains (p20 and p12) at its C-terminus. The short c-FLIP isoforms, c-FLIP_S_ and c-FLIP_R_, block DR-induced apoptosis by inhibiting procaspase-8 activation at the DED filament and at the DISC^19,20^. c-FLIP_L_ at the DISC can act both in a pro- and anti-apoptotic manner.

The pro-apoptotic role of c-FLIP is mediated by the formation of the procaspase-8/c-FLIP_L_ heterodimer in which the active center of procaspase-8 is stabilized in the active conformation through interactions with c-FLIP_L_, leading to the enhancement of the catalytic activity of the caspase-8 enzyme^21–23^. The pro-apoptotic role of c-FLIP_L_ strictly depends upon its amounts at the DISC and subsequently upon the number of the formed procaspase-8/c-FLIP_L_ heterodimers^24^. Upon intermediate levels of c-FLIP_L_ in the DED filaments, procaspase-8/c-FLIP_L_ heterodimers promote caspase-8 activity. Upon high concentrations of c-FLIP_L_, it plays only an anti-apoptotic role because high amounts of c-FLIP_L_ in the DED filaments downmodulates caspase-8 activation^19^. To specifically enhance pro-apoptotic effects of the caspase-8/c-FLIP_L_ heterodimer in cancer cells we have rationally designed the small molecule termed FLIPinB/FLIPinBγ targeting caspase-8-p43/c-FLIP_L_ heterodimer, the processed form of procaspase-8/c-FLIP_L_ heterodimer^25^. FLIPinB/FLIPinBγ binds at the interface of caspase-8/c-FLIP_L_ heterodimer and enhances its catalytic activity and thereby CD95L/TRAIL-induced cell death (Fig. 1B). FLIPinB is a small molecule discovered by virtual screening, while FLIPinBγ is a water-soluble analogue of FLIPinB^25^.

To investigate whether we can enhance the action of FLIPinB/FLIPinBγ via simultaneous targeting of intrinsic and extrinsic apoptosis pathways, the co-treatment with CD95L, the pharmacological inhibitors of anti-apoptotic Bcl-2 proteins and FLIPinB/FLIPinBγ was investigated (Fig. 1A). This co-treatment more efficiently induced the loss of cell viability, increased caspase-3/7 activation and apoptosis compared to the individual treatments. Taken together, we have demonstrated the possibilities for combinatorial treatment with FLIPinB/FLIPinBγ and pharmacological inhibitors of the intrinsic pathway.

## Methods

### Cell lines

HeLa-CD95 cells (HeLa cells with stable overexpression of CD95) and HeLa-CD95-FL cells (HeLa cells with stable overexpression of CD95 and c-FLIP_L_)^19,25^ were maintained in DMEM/Ham’s (Merck Millipore, Germany) supplemented with 10% heat-inactivated fetal calf serum, 0.0001% Puromycin and 1% Penicillin–Streptomycin in 5% CO_2_.

### Antibodies and reagents

The following antibodies were used for Western Blot analysis: polyclonal anti-caspase-3 antibody (#9662), polyclonal anti-PARP1 antibody (#9542), monoclonal anti-RIPK1 XP antibody (#3493), polyclonal anti-actin antibody (A2103; Sigma-Aldrich, Germany), polyclonal anti-mCherry antibody (ab183628, Abcam, UK), monoclonal anti-Bcl-2 antibody (sc-7382, Santa Cruz, USA), monoclonal anti-Bcl-x_L_ antibody (610209, BD, Germany), polyclonal anti-Bid antibody (#637, Cell Signaling, USA), monoclonal anti-Bak antibody (#578, Cell Signaling, USA), monoclonal anti-Bax antibody (#581, Cell Signaling, USA) and polyclonal anti-Mcl-1 antibody (sc-819, Santa Cruz, USA). Horseradish peroxidase-conjugated goat anti-mouse IgG1,-2a,-2b, goat anti-rabbit and rabbit anti-goat were from Santa Cruz (California, USA). All chemicals were of analytical grade and purchased from Merck (Darmstadt, Germany) or Sigma (Germany). Recombinant LZ-CD95L was produced as described^24^. The small molecules ABT-199 (A12500-10) and ABT-263 (A10022-10) were from Hölzel Diagnostika (Germany). The S63845 was from APExBIO (A8737, USA). FLIPinB was from Ambinter (Amb1202053, France). FLIPinBγ was produced as described^25^.

### Analysis of total cell lysates by Western Blot and protein quantification

The Western Blot analysis of total cellular lysates was performed in accordance to our previous reports^26^.

### Cell viability quantification by ATP assay

1.2 × 10^4^ cells per well were seeded the day before stimulation in 96 well plates. 50 µL of the CellTiter-Glo solution was added to each well. ATP content has been measured according to manufacturer’s instructions (Cell Titer-Glo-Luminescent Cell Viability Assay, Promega, Germany). The luminescence intensity was analyzed by a microplate reader Infinite M200pro (Tecan, Switzerland). The value of non-treated cells was taken as 100%. Every condition was performed in duplicate.

### Caspase-3/7 activity assay

1.2 × 10^4^ cells per well were seeded the day before stimulation in 96 well plates. 50 µL of the Caspase-Glo 3/7 solution was added to each well. Caspase activity was measured according to manufacturer’s instructions (Caspase-Glo 3/7 Assay, Promega, Germany). The luminescence intensity was analyzed by a microplate reader Infinite M200pro (Tecan, Switzerland). The caspase activity of non-treated cells was taken as one relative unit (RU). Every condition was performed in duplicate.

### Annexin-V-FITC and Sytox Orange staining

7.5 × 10^5^ HeLa-CD95-FL cells were seeded in six well plates the day before stimulation. Cells were stimulated with 1600 ng/mL CD95L, 50 µM FLIPinB and 10 µM ABT-263 or 1 µM S63845. Additionally 1 × 10^6^ HeLa-CD95-FL cells were used for heat shock as a positive control (56 °C, 10 min). The cells were harvested as in our previous reports described^27^. The cells were stained with 2 µL Annexin-V-FITC (Immuno Tools) and 0.75 µM Sytox Orange (ThermoScientific) in 70 µL Annexin binding buffer (BioLegend) per sample.

### Cell death analysis by imaging flow cytometry

The stained cells were immediately measured with the imaging flow cytometer (AMNIS FlowSight, Merck Millipore). For measuring the 488 nm excitation laser was used. Doublets and debris were gated out. 15,000 cells were analyzed and a compensation matrix was created to eliminate false positive results. For analysis the IDEAS version 6.0 was used. The cells were gated in double-negative (viable cells), Annexin-V-FITC positive (early apoptotic cells) and Annexin-V-FITC/Sytox Orange positive (late apoptotic cells) cell populations. The heat shock treatment was used as a positive control.

### Statistical analysis

For statistical analysis GraphPad prism 8 software was used. ANOVA Post Hoc Tukey tests were used. The following values were used: ****p < 0.0001; ***p < 0.0005; **p < 0.005; *p < 0.05; *ns* not significant.

## Results

### CD95L/FLIPinB/S63845 and CD95L/FLIPinB/ABT-263 co-treatment enhances the loss of cell viability of HeLa-CD95-FL cells

HeLa-CD95-FL cells are characterized by a high stable expression of c-FLIP_L_ proteins and were described by us earlier^19^. Upon high expression, c-FLIP_L_ inhibits CD95-mediated caspase activation and apoptosis. In line with this, the dose-dependent analysis of cell viability loss of parental HeLa-CD95 and HeLa-CD95-FL cells upon CD95L treatment has shown that HeLa-CD95-FL cells are more resistant towards CD95L stimulation compared to HeLa-CD95 cells^19^. In previous reports it was shown that administration of FLIPinB/FLIPinBγ in HeLa-CD95-FL cells slightly enhanced the CD95L-induced loss of cell viability^25^. Hence, in the current study we have selected this cell line as a model of CD95-resistant cell line to investigate the combinatory effects of FLIPinB/FLIPinBγ and the pharmacological inhibitors of anti-apoptotic Bcl-2 proteins. First, we characterized this cell line with respect to the expression of key Bcl-2 family members via Western Blot (Fig. 1C). The key Bcl-2 family members: Mcl-1, Bcl-2 and Bcl-x_L_ were expressed in HeLa-CD95-FL cells (Fig. 1C). Additionally, there are no differences in the expression level of Bcl-2 family members between HeLa-CD95 cells and HeLa-CD95-FL cells.

Mcl-1 is one of the key targets in contemporary anti-cancer research along with Bcl-2 and Bcl-x_L_^13^. Recently, a major progress has been achieved with the emergence of chemical inhibitors targeting Mcl-1 such as S63845 as well as ABT-263 and ABT-199 that target Bcl-2, Bcl-x_L_ and Bcl-w^10^. Next we tested whether we can enhance the action of FLIPinB via simultaneous targeting of c-FLIP_L_ and Mcl-1 or Bcl-2/Bcl-x_L_ in HeLa-CD95-FL cells.

**Figure 2:**
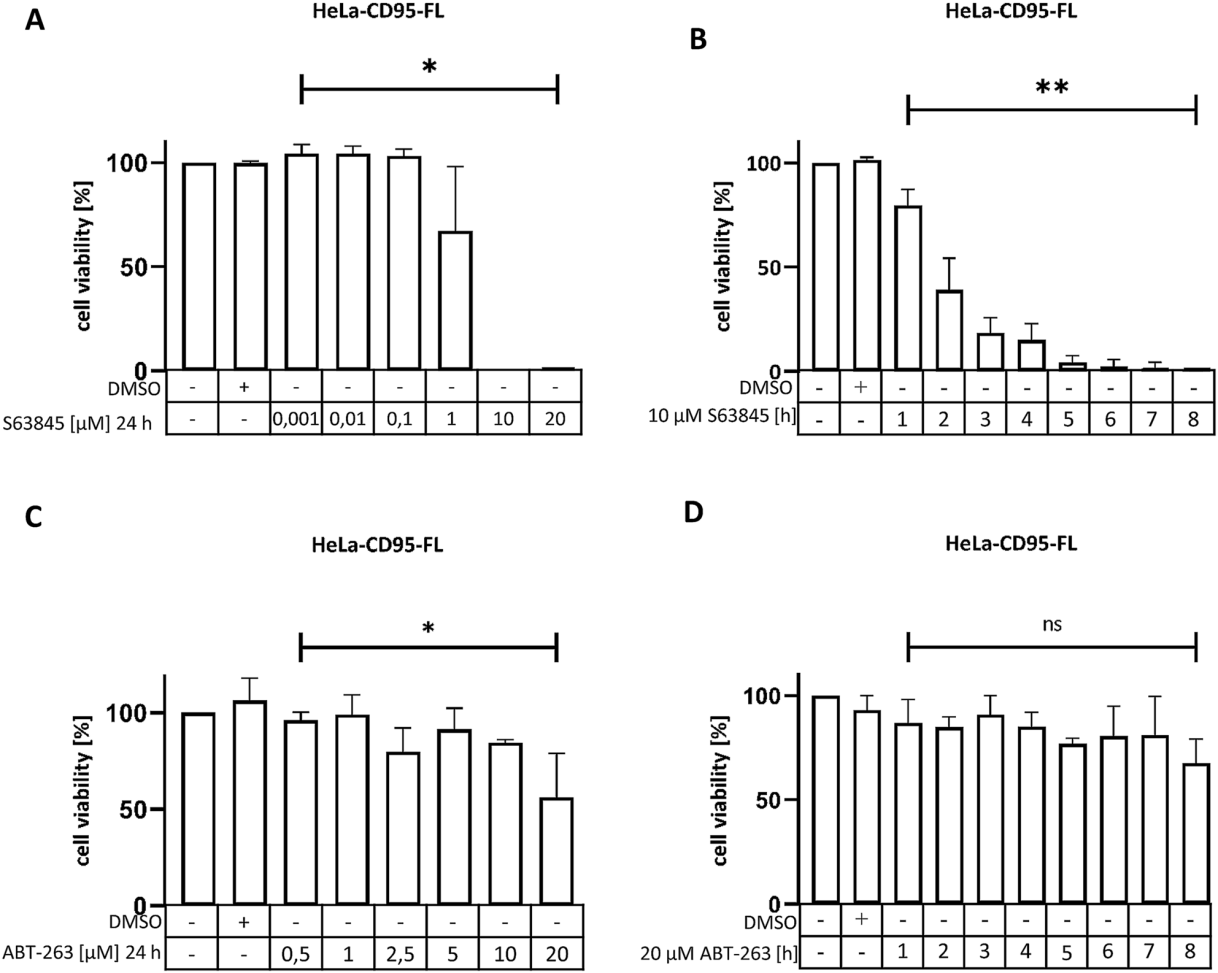
Treatment of HeLa-CD95-FL cells with S63845 and ABT263 leads to a loss of cell viability in a time- and dose- dependent manner. (**A**, **C**) HeLa-CD95-FL cells were stimulated with the indicated concentrations of S63845 (**A**) or ABT-263 (**C**) over 24 h (**B**,** D**). HeLa-CD95-FL cells were treated for 8 h every hour with 10 µM S63845 (**B**) or 20 µM ABT-263 (**D**). The cell viability was measured using the Cell Titer-Glo-Luminescent Cell Viability Assay. The value of unstimulated cells was taken as 100%. Mean and standard deviation are shown (n = 3). For statistical analysis ANOVA Post Hoc Tukey tests were used. ****p < 0.0001; ***p < 0.0005; **p < 0.005; *p < 0.05. *ns* not significant.

At the first step, S63845, ABT-263 and ABT-199 were administered to HeLa-CD95-FL cells in a dose- and time-dependent manner (Fig. 2, Supplementary Fig. 1A,B). The cell viability loss of HeLa-CD95-FL cells was observed upon increase of S63845 concentration and longer time intervals (Fig. 2A,B). In particular, the treatment with 10 µM of S63845 resulted in 100% loss of cell viability within 24 h after stimulation and treatment with 1 µM of S63845 led to 30% loss of cell viability within the same time interval. The treatment with ABT-263 also induced time- and dose-dependent cell viability loss in HeLa-CD95-FL cells, while the treatment with ABT-199, surprisingly, has shown hardly any effects (Fig. 2C,D, Supplementary Fig. 1A,B). The latter might be due to the lower expression levels of Bcl-2 in HeLa cells. Moreover, the effects of ABT-263 were less prominent compared to the same concentrations and time intervals for the treatment with the inhibitor of Mcl-1, S63845, on HeLa-CD95-FL cells (Fig. 2). Collectively, S63845 has triggered a stronger loss of viability upon its administration to HeLa-CD95-FL cells compared to ABT-263 and especially to ABT-199. Hence, for subsequent combinatorial treatment we have selected S63845 and ABT-263.

**Figure 3:**
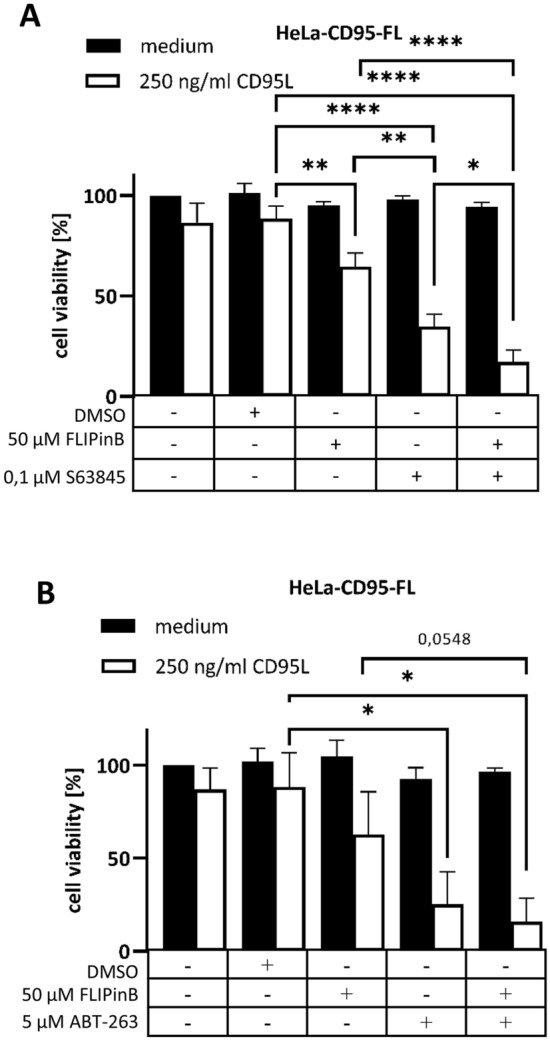
Combined treatment with FLIPinB, S63845/ABT-263 and CD95L enhances cell viability loss in HeLa-CD95-FL cells. (**A**, **B**) HeLa-CD95-FL cells were pretreated with the indicated concentrations of FLIPinB and S63845 (**A**) or ABT-263 (**B**) for 2 h. Afterwards the cells were stimulated with 250 ng/mL CD95L for 6 h. The cell viability was measured using the Cell Titer-Glo-Luminescent Cell Viability Assay. The value of unstimulated cells was taken as 100%. Mean and standard deviation are shown (n = 3). For statistical analysis ANOVA Post Hoc Tukey tests were used. ****p < 0.0001; ***p < 0.0005; **p < 0.005; *p < 0.05. *ns* not significant.

Next, HeLa–CD95-FL cells were treated with CD95L, S63845, or ABT-263 and FLIPinB/FLIPinBγ. The co-treatment CD95L/FLIPinB or CD95L/FLIPinBγ had moderate effects on the viability of HeLa-CD95-FL cells with the selected concentrations of CD95L and FLIPinB/FLIPinBγ (Fig. 3, Supplementary Fig. 1C). The co-treatment with CD95L/S63845 sensitized HeLa-CD95-FL cells towards CD95L treatment. The co-stimulation with CD95L, S63845 and FLIPinB/FLIPinBγ led to more prominent cell viability loss compared to the double treatments (Fig. 3A, Supplementary Fig. 1C). In particular, treatment with S63845 together with CD95L/FLIPinB or CD95L/FLIPinBγ enhanced a cell viability loss in HeLa-CD95-FL cells compared to CD95L/FLIPinB or CD95L/FLIPinBγ co-stimulations.

The similar tendency was observed for ABT-263 co-stimulation with CD95L/FLIPinB of HeLa-CD95-FL cells (Fig. 3B). However, this was observed upon higher concentrations of ABT-263 compared to S63845. Taken together, these results indicated that simultaneous targeting of c-FLIP_L_ and Mcl-1 as well as Bcl-2/Bcl-x_L_ efficiently induces the loss of cell viability.

### CD95L/FLIPinB/S63845 and CD95L/FLIPinB/ABT-263 co-treatment sensitizes the cells towards apoptotic cell death

**Figure 4:**
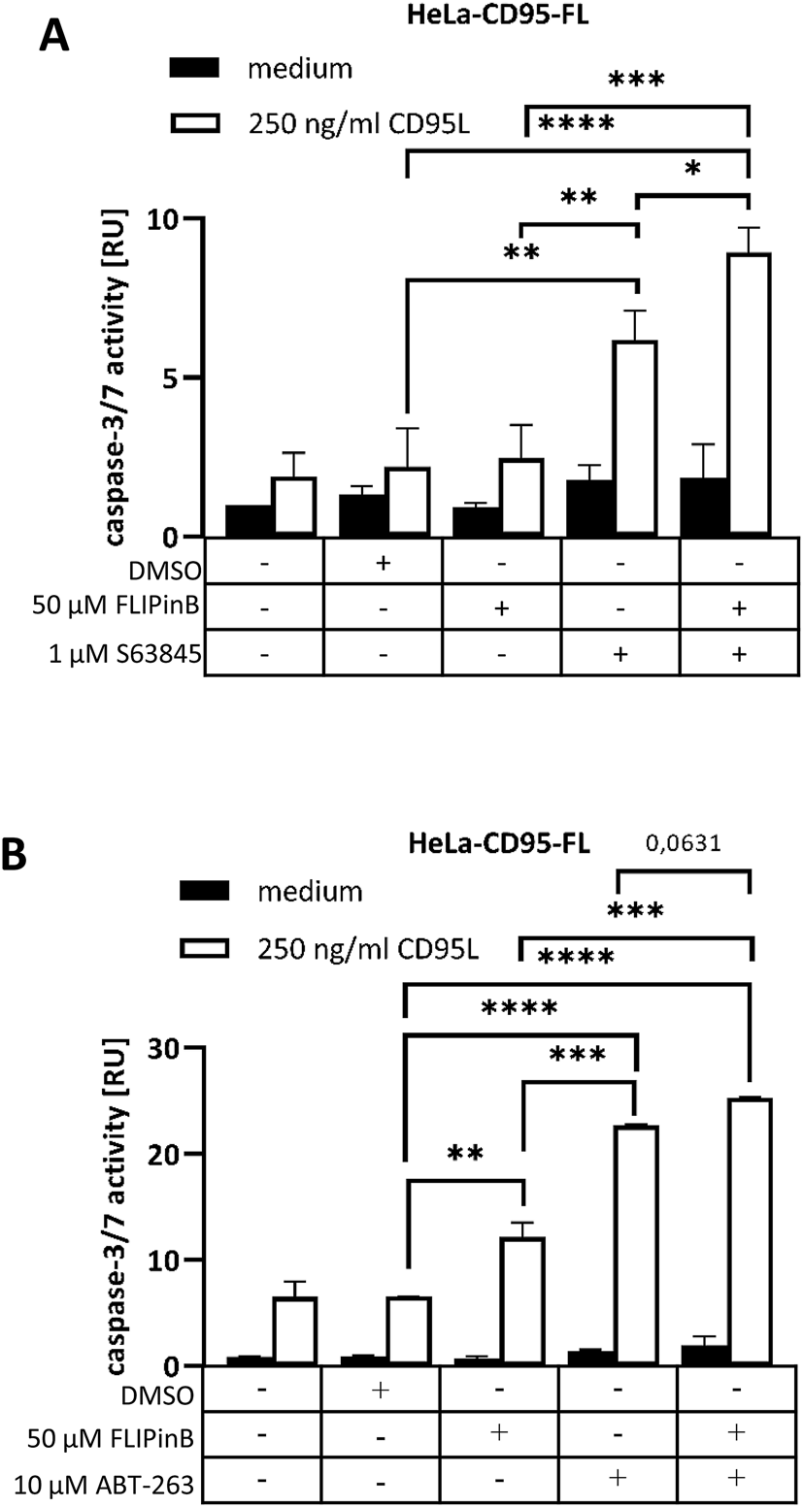
Co-treatment with FLIPinB, S63845/ABT-263 and CD95L leads to increased caspase activity in HeLa-CD95-FL cells. (**A**, **B**) HeLa-CD95-FL cells were pretreated with the indicated concentrations of FLIPinB and S63845 (**A**) or ABT-263 (**B**) for 2 h. Subsequently, the cells were treated for 2 h with CD95L. Caspase-3/7 activity was measured using the Caspase-Glo 3/7 Assay. The value of  unstimulated cells was taken as 1 RU (relative unit). Mean and standard deviation are shown (n = 3) (**A**) or (n = 2) (**B**). For statistical analysis ANOVA Post Hoc Tukey tests were used. ****p < 0.0001; ***p < 0.0005; **p < 0.005; *p < 0.05. *ns* not significant.

To validate whether the cells upon CD95L/FLIPinB/S63845 or CD95L/FLIPinB/ABT-263 co-treatment undergo apoptotic cell death caspase-3/7 activity assays were carried out. CD95L/S63845 or CD95L/ABT-263 co-treatment led to an increase in caspase-3/7 activity compared to CD95L-only treatment in HeLa-CD95-FL cells. Furthermore, a stronger increase in caspase-3/7 activity upon CD95L/FLIPinB/S63845 or CD95L/FLIPinB/ABT-263 co-stimulation compared to CD95L/FLIPinB, CD95L/ABT-263 or CD95L/S63845 treatment was detected (Fig. 4). To further validate the observed effects via independent approach we have analyzed caspase processing using Western Blot analysis (Supplementary Fig. 2). Western Blot analysis demonstrated that S63845 slightly increased CD95L/FLIPinB- or CD95L/FLIPinBγ-induced RIPK1, PARP1 and procaspase-3 processing (Supplementary Fig. 2). Collectively, these results demonstrate that S63845 and ABT-263 enhance CD95L/FLIPinB or CD95L/FLIPinBγ-induced caspase activation and act on the apoptotic branch of the pathway.

**Figure 5:**
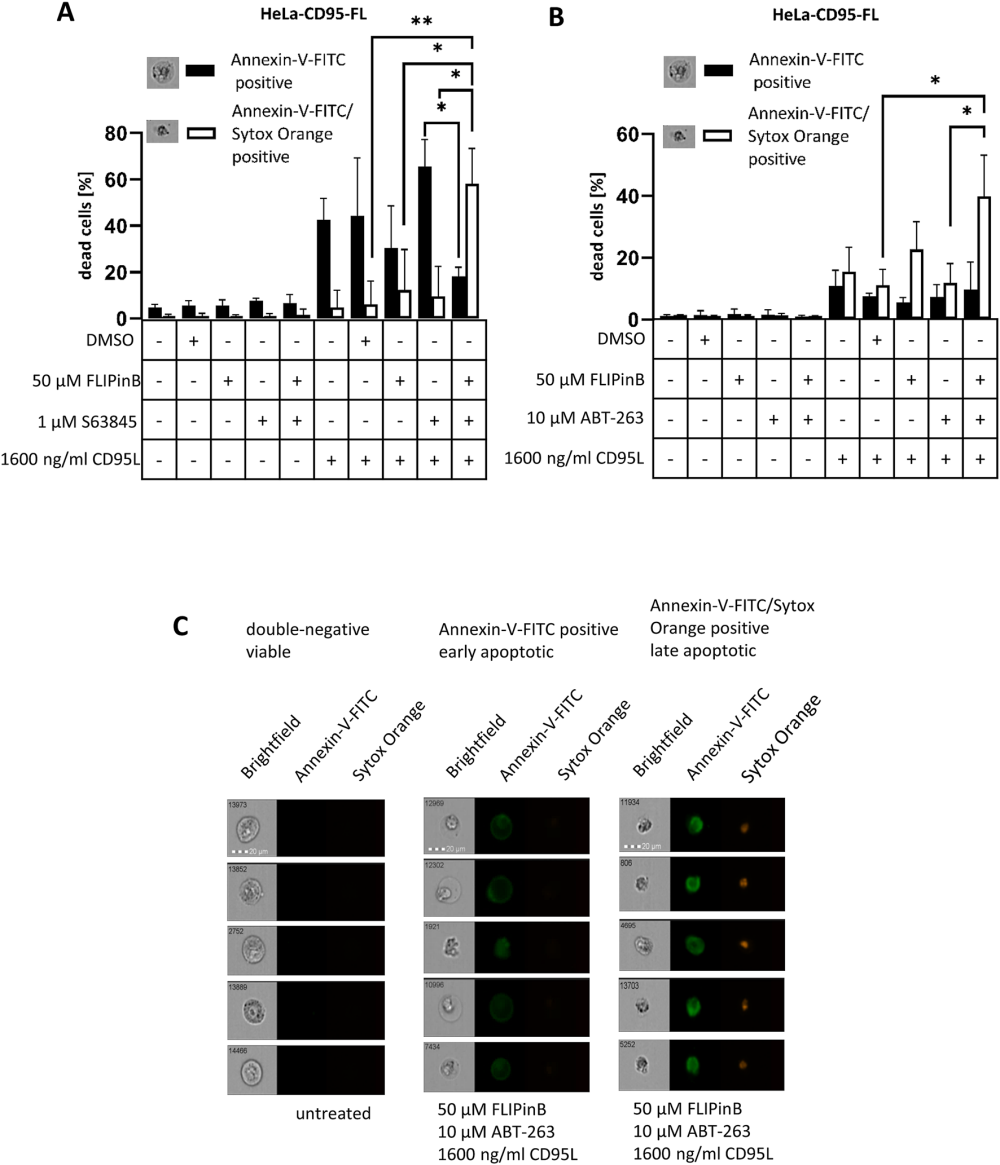
CD95L/S63845/FLIPinB and CD95L/ABT-263/FLIPinB co-treatments lead to an increase of late apoptotic cells in HeLa-CD95-FL cells. (**A**, **B**) HeLa-CD95L-FL cells were pretreated for 2 h with 50 µM FLIPinB and 1 µM S63845 (**A**) or 10 µM ABT-263 (**B**) and subsequently stimulated with 1600 ng/mL CD95L for 4 h. The samples were stained with Annexin-V-FITC and Sytox Orange and gated for Annexin-V-FITC/Sytox Orange double positive (late apoptotic) and Annexin-V-FITC positive (early apoptotic) cells using AMNIS FlowSight. Mean and standard deviation are shown (n = 3). The representative images (bright field channel) for Annexin-V-FITC-positive and Annexin-V-FITC/Sytox Orange double positive cells are shown. (**C**) Representative pictures of the treated cells. Cells are divided into double-negative (viable), Annexin-V-FITC single positive and Annexin-V-FITC/Sytox Orange double-positive cells. Five cells per condition are shown. Pictures for double-negative cells were taken from unstimulated cells while Annexin-V-FITC and Annexin-V/Sytox Orange positive cells are taken from CD95L/FLIPinB/ABT-263 treatment. For statistical analysis ANOVA Post Hoc Tukey tests were used. ****p < 0.0001; ***p < 0.0005; **p < 0.005; *p < 0.05; *ns* not significant.

To further confirm whether upon CD95L/FLIPinB/S63845 or CD95L/FLIPinB/ABT-263 co-treatment HeLa-CD95-FL cells undergo apoptotic cell death, we used the developed by us approach based on the imaging flow cytometry^28^. This method uses the combination of the analysis of Annexin-V-FITC staining and the nuclear morphology of apoptotic cells. In particular, nuclei of late apoptotic cells are sharp, therefore, upon staining with Sytox Orange the late apoptotic nuclei are typically observed as spots of bright Sytox Orange-intensity (Fig. 5). The application of Annexin-V-FITC/Sytox Orange staining followed by imaging flow cytometry analysis allowed to observe the increase in the amounts of apoptotic cells upon both CD95L/FLIPinB/S63845 or CD95L/FLIPinB/ABT-263 treatment compared to CD95L treatment (Fig. 5, Supplementary Figs. 3, 4). This demonstrates that the combination of FLIPinB and Bcl-2 family antagonists/Mcl-1 inhibitors enhances CD95L-mediated apoptosis.

## Discussion

Targeting simultaneously intrinsic and extrinsic pathway provides a broad band of therapeutic options in anti-cancer therapies. In particular, the strength of our study is that it combines selective inhibitors for the key components of intrinsic and extrinsic apoptosis pathways, which might allow to avoid cytotoxicity induced by the conventional therapeutic approaches.

The parental HeLa-CD95 cells were used by several groups in the previous studies and shown to be highly sensitive to CD95 stimulation, form high levels of CD95 DISC and undergo cell death. In our study we have used HeLa-CD95-FL cells as a cell line, which is resistant to CD95L-induced apoptosis. HeLa-CD95-FL cells are characterized by a high overexpression of c-FLIP_L_ and therefore they are more resistant to CD95-induced cell death compared to parental HeLa-CD95 cells. Hence, this cell line presents a suitable cell model for the sensitization by combinatorial treatment^19^. Recently, using quantitative mass spectrometry, it was demonstrated that the high overexpression of c-FLIP_L_ in HeLa-CD95-FL cells leads to the formation of the DISC comprising short DED filaments with the ratio between c-FLIP_L_ and caspase-8 of 1 to 1^19^. The structural modeling has demonstrated that under these conditions there is a possibility for the formation of the caspase-8 homodimers though it is very limited. This results in the limited apoptosis induction upon CD95 stimulation in HeLa-CD95-FL cells. Accordingly, HeLa-CD95-FL cell line can be used as a model for type II cells with the reduced level of caspase-8 activation and apoptosis induction via CD95 and at the same time high level of CD95 expression.

FLIPinB/FLIPinBγ targets caspase-8/c-FLIP_L_ heterodimer and its effects strictly depend upon the number of the heterodimers formed at the DISC. Accordingly, upon the low levels of c-FLIP_L_, no action of this compound can be expected due to the low number of the formed heterodimers. Upon intermediate levels of c-FLIP_L_ in the DED filaments, the action of FLIPinB/FLIPinBγ is expected to be stronger and with high concentrations of c-FLIP_L_, the amounts of heterodimers at the DED filaments will be further decreased, downmodulating the action of FLIPinB. To account for this complexity, we have developed a mathematical model that allows to predict the action of FLIPinB on enhancement of procaspase-8 processing at the DISC in the various cellular landscapes^25^. This quantitative analysis has to be taken into consideration for the development of future therapeutic approaches.

The combination of targeting both extrinsic and intrinsic pathways were suggested before in a number of studies^29^. In particular, simultaneous targeting of c-FLIP and Mcl-1 has been considered as a promising strategy for anti-cancer research^30–32^. However, in most of the previous studies this involves the non-specific downregulation of c-FLIP and Mcl-1, which has a number of disadvantages. In our study we used the first-in-class chemical probe targeting c-FLIP_L_ and the specific inhibitor of Mcl-1, which makes our approach more specific^25^. The future studies aimed on improving the structure of FLIPinB should allow to further increase the effects of this combinatorial treatment. Interestingly, the best synergism was found between the co-treatment of c-FLIP_L_ and Mcl-1 inhibitors. The latter worked in lower concentrations much better than Bcl-2 antagonists. This might be due to the higher expression of Mcl-1 compared to Bcl-2 and Bcl-xL in the selected cellular models.

The drugs targeting intrinsic apoptosis pathway are already known for a long time. However, in contrast to the major regulators of the intrinsic apoptosis pathway, to which specific small molecule-based inhibitors have already been successfully developed, targeting core components of extrinsic pathway is only starting to be addressed^12^. This is because the 3D structures of the major initiatory proteins of the pathway have only been solved recently^5^. This, in particular, concerns the major proteins of the DISC and the 3D structure of the CD95 DISC itself. Solving of the latter structure would undoubtedly open new possibilities for drug discovery and the development of novel, more effective and selective anti-cancer therapies.

Taken together, this work provides a basis for the development of new therapies against cancer via specific enhancement of the activity of the key enzyme of the extrinsic apoptosis-caspase-8 and Bcl-2 family members, acting simultaneously on extrinsic and intrinsic apoptosis pathways, thereby paving new avenues for specifically inducing apoptosis.

## Supplementary information

Supplementary Figures.
