# Supplementary material for: Pharmacological targeting of c-FLIPL and Bcl-2 family members promotes apoptosis in CD95L-resistant cells
Source: Sci Rep. 2020 Nov 30;10:20823. doi: 10.1038/s41598-020-76079-1 (PMC7705755; doi:10.1038/s41598-020-76079-1)
Supplement: Supplementary file 1 — Supplementary Figures. [file 41598_2020_76079_MOESM1_ESM.pdf]

Pharmacological targeting of c-FLIP$_L$ and Bcl-2 family members promotes apoptosis in CD95L-resistant cells

Corinna König[1*], Laura K. Hillert-Richter[1*], Nikita V. Ivanisenko[2], Vladimir A. Ivanisenko[2], Inna N. Lavrik[1#]

[1-]Translational Inflammation Research, Medical Faculty, Otto von Guericke University Magdeburg, Magdeburg, 39106, Germany

[2-]The Federal Research Center Institute of Cytology and Genetics, The Siberian Branch of the Russian Academy of Sciences, Prospekt Lavrentyeva 10, Novosibirsk, 630090, Russia

*- equal contribution
[#-] corresponding author/lead contact

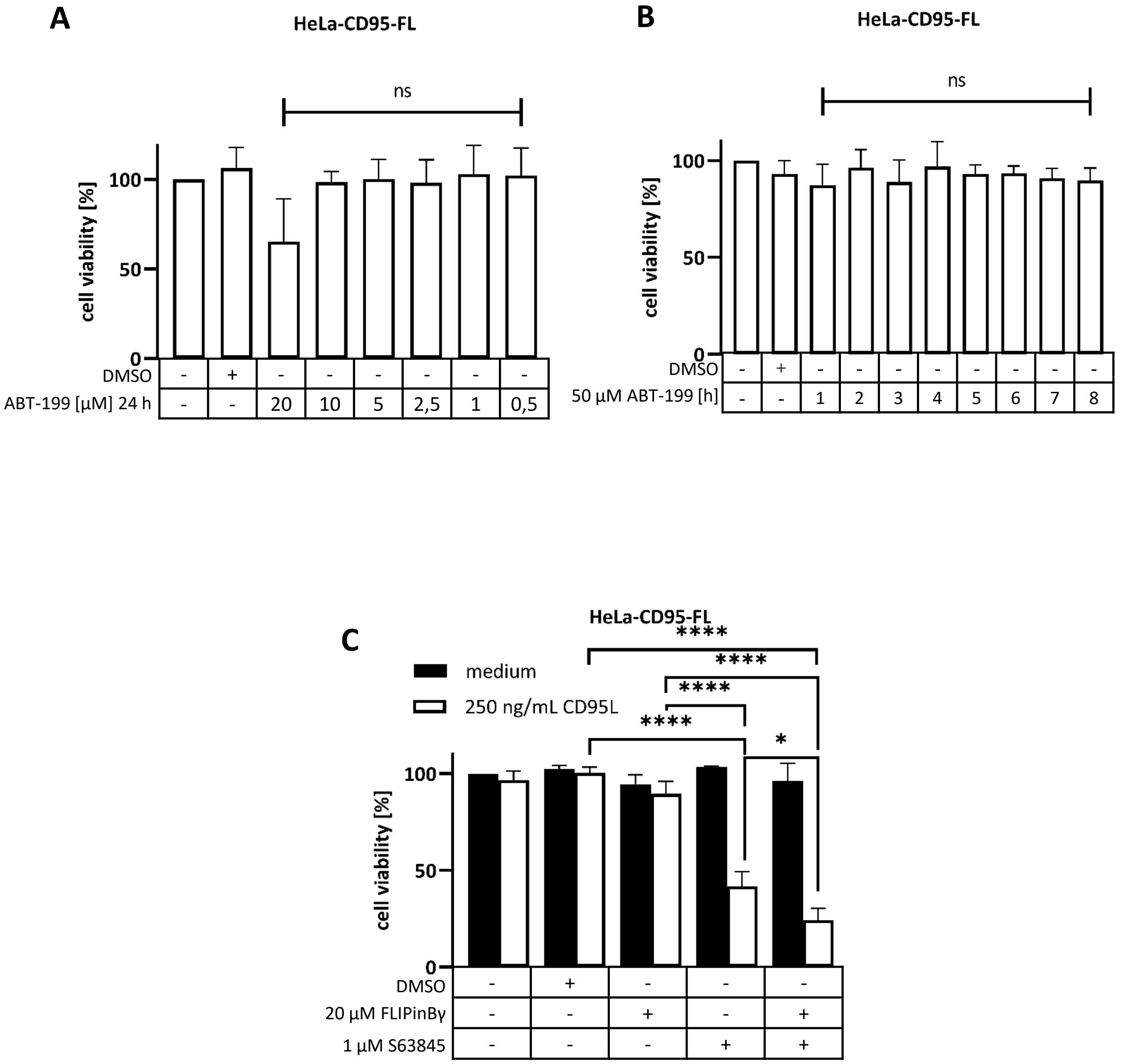

Supplementary Figure 1: Co-treatment with ABT-199 and CD95L/S63845/FLIPinBγ of HeLa-CD95-FL cells

A, B) HeLa-CD95-FL cells were stimulated with the indicated concentrations of ABT-199 over 24 h (A) or for 8 h with 50 µM ABT-199 (B). **C)** HeLa-CD95-FL cells were treated with 20 µM of FLIPinBγ and 1 µM S63845 for 2 h. Afterwards the cells were treated with CD95L for 6 h. The cell viability was measured using the Cell Titer-Glo-Luminescent Cell Viability Assay. Unstimulated cells were taken as 100 %. Mean and standard deviation are shown (n=3). For statistical analysis ANOVA Post Hoc Tukey tests were used. ****p<0,0001; ***p<0,0005; **p<0,005; *p<0,05; ns: not significant

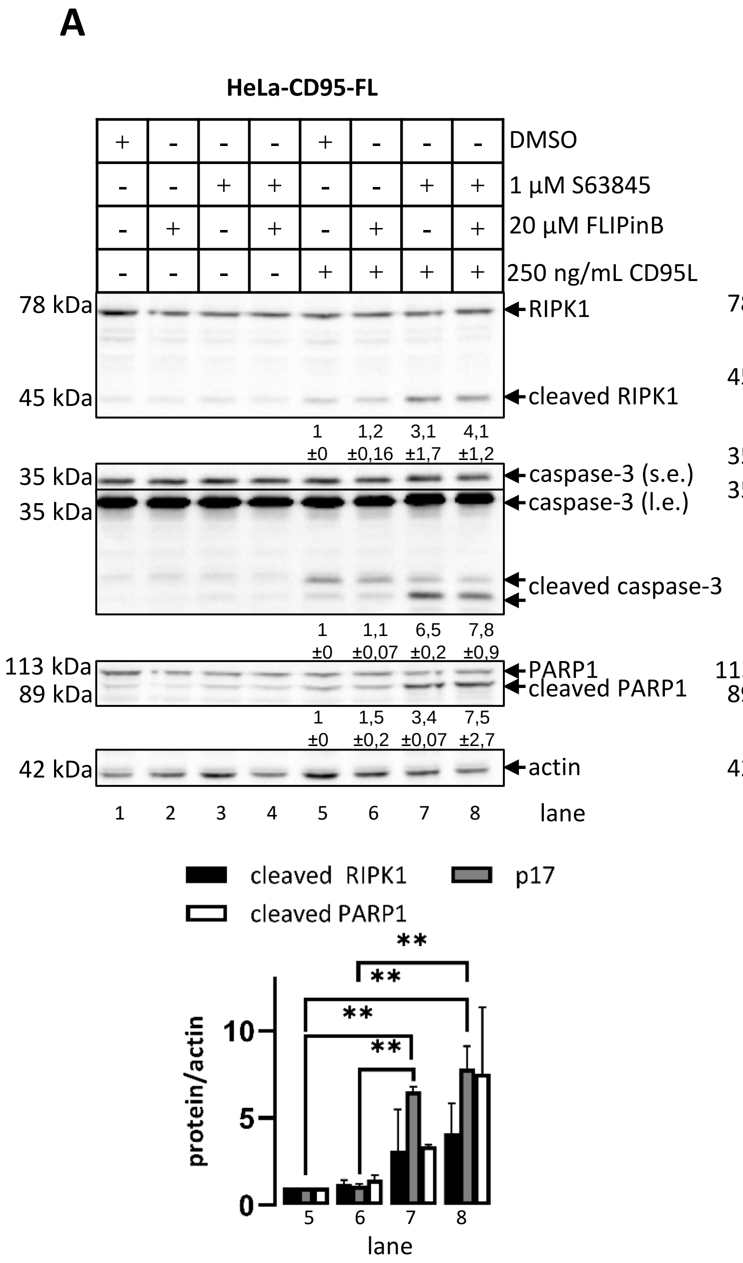

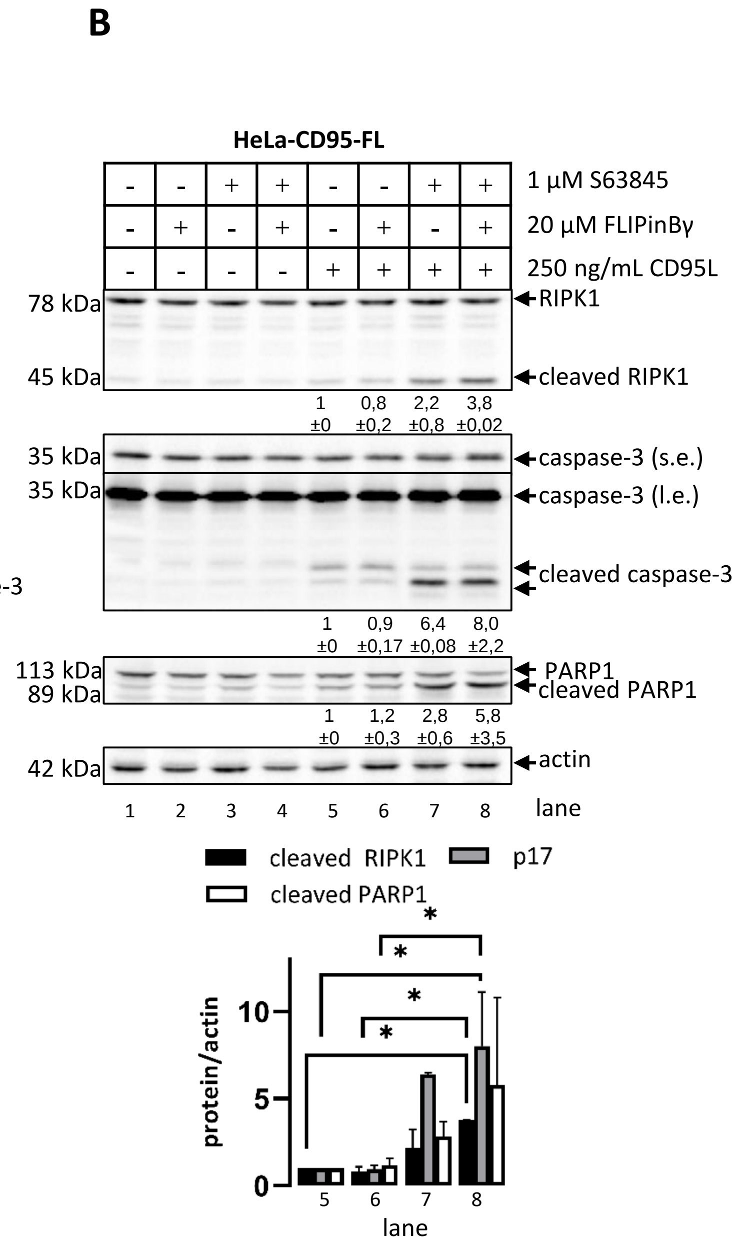

Supplementary Figure 2: Co-treatment leads to an increase of cleavage products of apoptotic key proteins.
A, B) HeLa-CD95-FL cells were pretreated for 2 h with the indicated concentrations of S63845 and FLIPinB (A) or FLIPinBγ (B). Afterwards the cells were stimulated with 250 ng/ml CD95L for 4 h. Total cell lysates were analyzed by Western Blot with the indicated antibodies. Actin served as a loading control. The Western Blot Quantification is shown with mean and standard deviation under the corresponding Western Blot and as diagrams below the Western Blot. (n=2). For statistical analysis ANOVA Post Hoc Tukey tests were used. ****p<0,0001; ***p<0,0005; **p<0,005; *p<0,05; ns: not significant

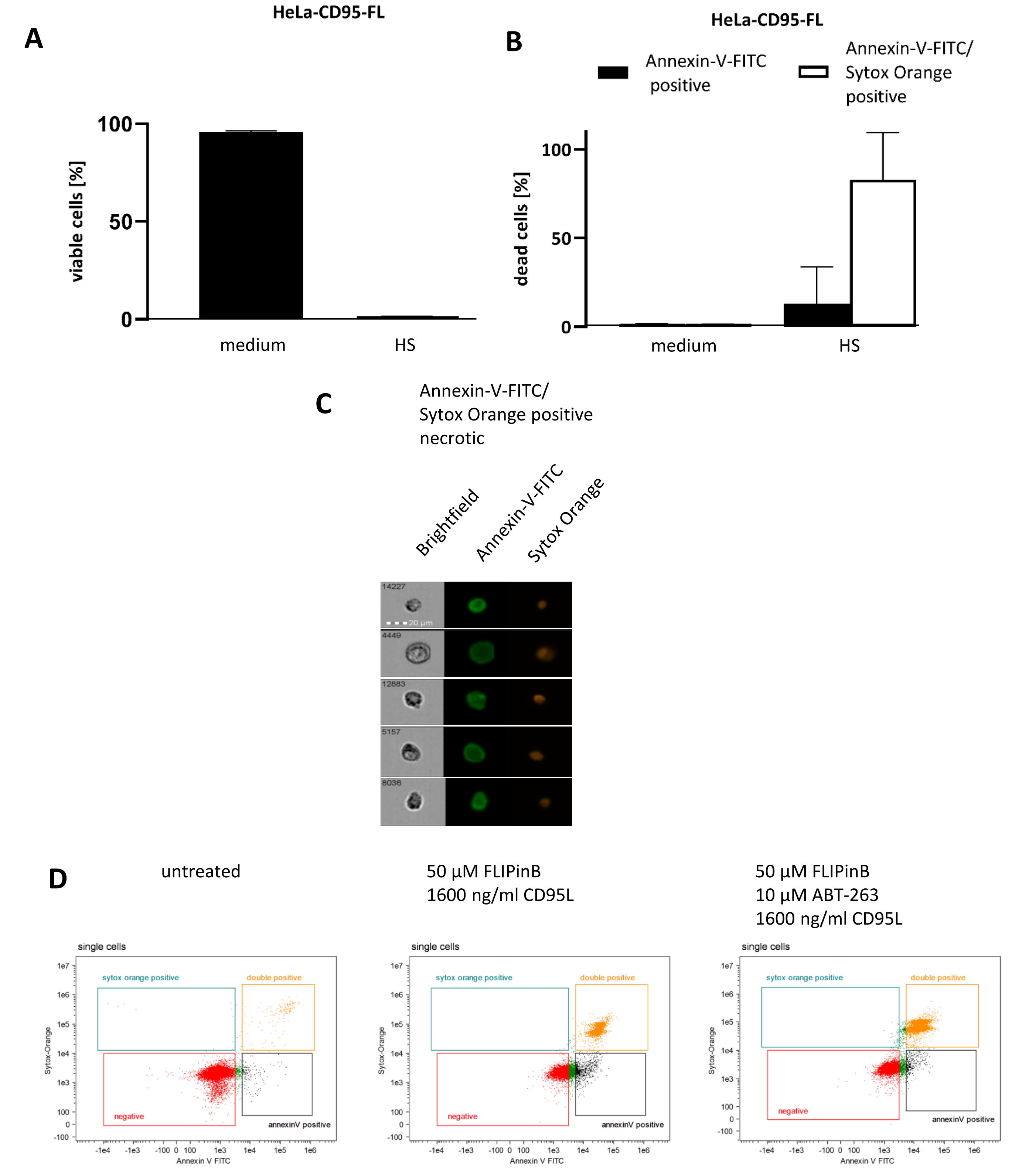

Supplementary Figure 3: Heat shock treatment was used as a positive control for CD95L/ABT-263/FLIPinB treatment of HeLa-FL cells, which have shown double positive staining

A) HeLa-CD95-FL cells were incubated for 10 min at 56°C for heat shock (HS). The unstimulated and heat shock-treated samples were measured using AMNIS FlowSight and gated for double-negative cells (viable cells) **B)** The HS samples were measured using AMNIS FlowSight and gated for Annexin-V-FITC/Sytox Orange positive and Annexin-V FITC cells. **C)** Representative pictures of the treated cells. Shown are Annexin-V-FITC/Sytox Orange positive cells. Mean, standard deviation and representative pictures are shown (n=3). **D)** The gating strategy is shown for double-negative, Annexin-V-FITC positive and Annexin-V-FITC/Sytox Orange positive populations after the indicated stimulation. The samples were measured using AMNIS FlowSight.

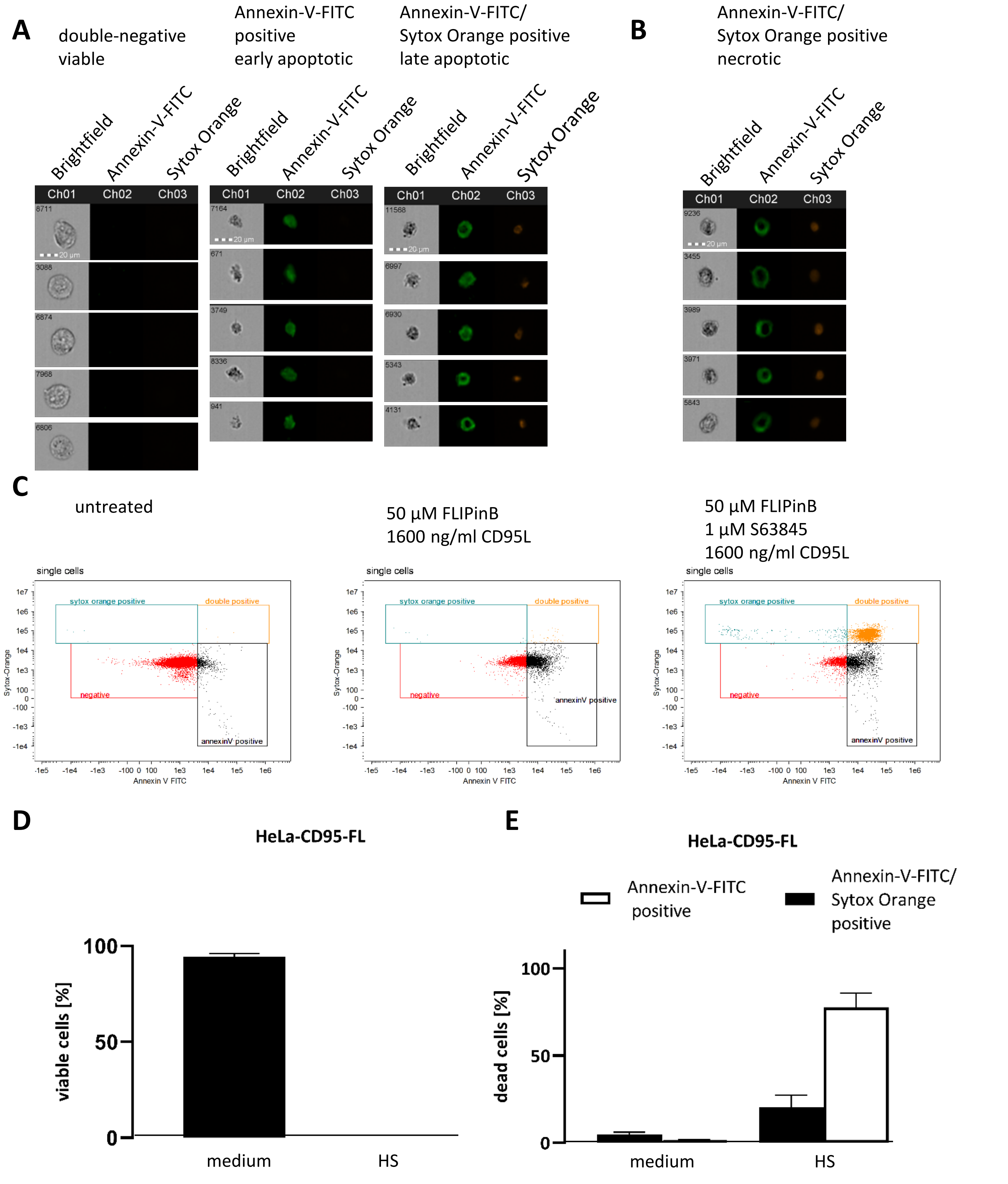

Supplementary Figure 4: Heat shock treatment used as a positive control for CD95L/S63845/FLIPinB treatment results in double positive staining of HeLa-CD95-FL cells.

A, B) Representative pictures of double-negative (viable), Annexin-V-FITC-positive and Annexin-V-FITC/Sytox Orange positive cells after treatment (A) or Annexin-V-FITC/Sytox Orange positive cells after HS (B) are shown. **C)** Shown is the gating strategy for double- negative, Annexin-V-FITC positive and Annexin-V-FITC/Sytox Orange positive populations after the indicated stimulation. Samples were measured with AMNIS FlowSight. **D)** HeLa-CD95-FL cells were incubated for 10 min at 56°C for heat shock (HS). Samples were measured using AMNIS FlowSight and gated for double-negative cells (viable cells). **E)** Unstimulated(medium) and HS samples were measured using AMNIS FlowSight and gated for Annexin-V-FITC/Sytox Orange positive and Annexin-V FITC cells.
